# A Rapid Decision Sampling Plan for Implementing Area—Wide Management of the Red Palm Weevil, *Rhynchophorus ferrugineus*, in Coconut Plantations of India

**DOI:** 10.1673/031.008.1501

**Published:** 2008-02-27

**Authors:** J. R. Faleiro, J. Ashok Kumar

**Affiliations:** Plant Protection Laboratory, ICAR Research Complex for Goa, Ela, Old Goa- 403 402, India

**Keywords:** sequential sampling, *Cocos nucifera*

## Abstract

The red palm weevil *Rhynchophorus ferrugineus* Olivier (Curculionidae/Rhynchophoridae/Dryophthoridae) is a lethal pest of young coconut palms, *Cocos nucifera* L. (Arecales: Arecaceae), with a highly aggregated population distribution pattern. *R. ferrugineus* is managed in several coconut growing countries using area-wide pheromone based programmes that need a substantial commitment of funds over a period of time. Often, decisions to implement area-wide management of *R. ferrugineus* are based on pheromone trap captures in surveillance traps and or infestation reports. Implementing area-wide management of this pest on the basis of such data can be inaccurate, as it may either under or over estimate the pest intensity in the field. This study presents sampling plans for rapid and accurate classification of *R. ferrugineus* infestation in coconut plantations of India by inspecting palms to detect infestation in a sequence until a decision to either implement or not to initiate area-wide management of *R. ferrugineus* can be made. The sampling plans are based on a common aggregation index of 3.45, assumed action threshold values of either 1.0 (plan A) or 0.5 (plan B) per cent infested palms and a risk factor of making the wrong decision set at 0.05. Using plans A and B, if the cummulative number of infested palms in a young 1 hectare coconut plantation is zero out of 150 palms for both plans, then area-wide management is not required, while on the other hand, if the cummulative number of infested palms for the same area is 6 (plan A), or 5 (plan B), then area-wide management of *R. ferrugineus* is essential. The proposed sampling plans are efficient tools in decision making, particularly at very low and high levels of infestation and can also be used to assess the performance of *R. ferrugineus* IPM programmes that are in progress. These plans not only save time and money as only a small area needs to be sampled to arrive at a correct decision, but are also efficient in rating the infestation level accurately.

## Introduction

Coconut, *Cocos nucifera* L. (Arecales: Arecaceae) is cultivated in 12.78 million hectares in 93 countries. In India the crop is grown in 1.93 million hectares and provides livelihood to over 10 million people (Mathew 2004). The red palm weevil, *Rhynchophorus ferrugineus* Olivier (Curculionidae/Rhynchophoridae/Dryophthoridae) a concealed tissue borer of palms, is reported from 15 per cent of the coconut growing countries mainly from tropical South and South-East Asia (Faleiro 2006a). Infested palms in the early stage of attack respond to treatment with insecticide, while palms in the late stage of attack often die (Abraham *et al*. 1998). Given that coconut is a high value crop and *R. ferrugineus* is a lethal pest, this crop-pest relationship warrants immediate action to control the pest. Faleiro (2006a, b) recommend an action threshold of 1% infested palms to initiate area-wide farmer participatory mamagement of *R. ferrugineus* in large plantations. Currently, it is managed by employing an integrated pest management (IPM) strategy comprised mainly of mass trapping adult weevils using ferrugineol-based food baited traps (Hallett et al. 1993), crop and field sanitation, preventive chemical treatments of wounds, treating palms infected with bud rot disease or infested with the coconut rhinoceros beetle, *Oryctes rhinoceros*, attack to prevent attraction of *R. ferrugineus* adults, filling frond axils of young palms with mixture of insecticide and sand, curative treatment of infested palms in the early stage of attack, eradicating severely infested palms, cutting fronds if required at a distance of one meter from the frond base, and educating and training farmers and agricultural officers about *R. ferrugineus*—IPM (Abraham and Kurian 1975; Abraham et al.1989).

This IPM programme was implemented in coconut plantations at several farms in endemic pockets of Sri Lanka and India (Rajapakse *et al*. 1998; Faleiro 2005). In date palm however, *R. ferrugineus* has been managed in many middle Eastern countries through area-wide IPM programmes implemented throughout the province/district and are larger as compared to the area-wide *R. ferrugineus*-IPM programmes implemented in coconut plantations of South Asia (Ezaby et al. 1998; Abraham et al. 2000; Vidhyasagar et al. 2000; Soroker et al. 2005). The superiority of area-wide IPM programmes over the conventional farm-by-farm management of insect pests has been previously recommended by many (Knipling 1992; Lindquist 1998; Mumford 1998; Yu and Leung 2006). Such IPM programmes consider the spatial and temporal distribution of the pest, are long term in nature and attempt to persistently reduce the pest population in a relatively large area to non-economic status (Lindquist 1998).

Before implementing area-wide management of *R. ferrugineus* in coconut plantations it is essential to accurately assess damage level in the field. The concept of sequential analysis proposed by Wald 1947 has been utilized in the past to classify infestation levels (Onsager 1976). Based on this concept, sequential sampling plans to rapidly classify infestation and accurately decide on initiating control measures have been developed for a diverse range of insect pests in several crops (Morris 1954; Waters 1955; Ives and Warren 1965; Suman and Wahi 1981; Rai et al. 1982; Shepard et al. 1986; Kumar 1996; Yu et al. 2005).

Here, two sequential sampling plans are presented for implementing area-wide management of *R. ferrugineus* in coconut where inspection of young palms to locate infestation by *R. ferrugineus* is done repeatedly until an accurate decision on implementing area-wide management of *R. ferrugineus* can be made. Plan A is based on an action threshold of 1% infested palms while plan B is developed at the lower action threshold of 0.5 % infestation.

## Materials and methods

As outlined by Morris (1954) the first step in the development of a sequential sampling plan is to establish the spatial distribution of the insect in nature. *R. ferrugineus* is known to follow a highly aggregated or clumped distribution, with a common clumping parameter (K) of 3.45 established previously by Faleiro et al. 2002 in coconut plantations of India, which is used for this study. K is a valid and readily computed measure of aggregation for a wide range of insect counts (Bliss and Owen 1958; Waters 1959). The sequential sampling plans developed here for initiating area-wide management of *R. ferrugineus* in coconut plantations of India are based on action threshold levels of 1% (plan A) and 0.5 % (plan B) infested palms. Although, Faleiro (2006a and b) proposed area-wide management of *R. ferrugineus* at 1% infestation, the high value of the crop and the lethal nature of the pest may warrant early action against *R. ferrugineus*. Hence, plan B of this article is developed to initiate area-wide management of *R. ferrugineus* at 0.5 % infested palms. Both sampling plans are developed at a risk factor of a and b set at 0.05, where a is the probability of recommending area-wide management when it is not required and b is the probability of failing to recommend area-wide management when required.

The acceptance and rejection lines for the hypothesis of “not implementing area-wide management of *R. ferrugineus*” are based on the sequential probability ratio test, SPRT formulae outlined by Wald (1947) and found in Southwood and Henderson (2000) as

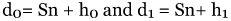

where, d_0_ and d_1_ are the cumulative maximum and minimum infested palms per hectare for not recommending and recommending area-wide management of *R. ferrugineus*, respectively and n is the area sampled in hectares. In India the general recommendation for planting coconut varies from 7.5 to 9.0 m in the square system of planting which accommodates a plant population varying from 123 to 178 palms per hectare (Thampan 1981). A palm density of 150 palms per hectare is assumed for the purpose of this study.

*R. ferrugineus* mostly attacks young palms below the age of 20 years (Nirula 1956), hence the number of palms sampled in the susceptible age group rather than the area covered would form the basis of sampling to detect *R. ferrugineus* infested palms.

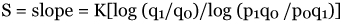
. where, K is the index of aggregation, p0 = m_0_/K, p1 = m_1_/K, q0 = p0+1 and q1 = p1+1 and m0 and mi are the lower and upper levels of the infestation set at 1/3 and 2/3 of the assumed action threshold levels. Usually, mi would correspond to the economic threshold, which is the level at which treatment should be initiated to prevent economic loss (Stern et al. 1959). Further, h0 = intercept of the lower line and is given by

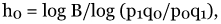
, where B= b/1- a and h_1_= the intercept of the upper line which is given by log A/log (p1q0/p0q1), where A= 1- b/a and a and b are the probabilities of failing to recommend the correct decision i.e. accepting and rejecting area-wide management when not required and required, respectively, which are set at 0.05 in these plans.

The operating characteristic (OC) and the average sample number (ASN) curves are helpful in visualizing the performance of the sequential sampling plan (Binns et al. 2000; Binns and Nyrop 1992). The OC and ASN curves were calculated based on the formulae outlined for negative binomial distribution by Oakland (1950) and Waters (1955). The OC curves for the two sampling plans in this investigation give the probability L(p) of not resorting to area-wide management of *R. ferrugineus* at various levels of infestation (p) and are derived from

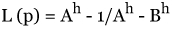

where, A and B are defined earlier and h is the dummy variable while p = 1- (q0 /q1)^h^ /(p1q0/p0q1)^h^ -1. Further, the ASN curve developed for plans A and B in this study indicate the number of samples at different levels of infestation and are given by

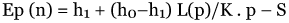

where h1, h0, L(p), K, p and S are defined above.

## Results and Discussion

**Figure 1.**
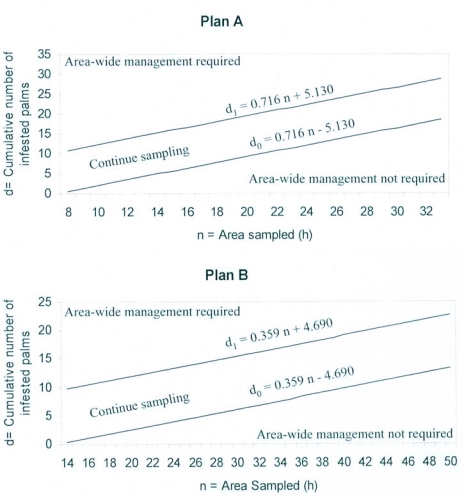
Sequential sampling plans for area-wide management of *Rhynchophorus ferrugineus* in coconut.

The two decision lines for rejecting or accepting the hypothesis of not implementing area-wide management of *R. ferrugineus* at an action threshold of 1% are presented in plan A as d0 = 0.716n - 5.130 and d1= 0.716n + 5.130 where d0 is the maximum value for the lower class and d_1_ the minimum value for the upper class in terms of cumulative number of palms infested and n is the area in hectares to be sampled. A stricter assumed action threshold of 0.5 % infested palms was also considered to work out decision lines for rejecting or accepting the above hypothesis and are presented under plan B as d0 = 0.359n - 4.690 and d1= 0.359n + 4.690, where d0, d1 and n have been explained above (Figure 1).

In other words, in plan A if d0 ≤ 0.716n - 5.130 then the infestation due to *R. ferrugineus* is rated as light and area-wide management is not required, while on the other hand if d_1_ ≥ 0.716n + 5.130, the infestation level is rated as high, warranting the need to implement area-wide management of *R. ferrugineus*. Similarly, under plan B, the equations to rate the infestation due to *R. ferrugineus* as light or high would be do ≤ 0.359n - 4.690 and d1 ≥ 0.359n + 4.690, respectively where d0, d1 and n are described above. Beginning in the 1950's sequential sampling plans have been developed to decide on the control of insect pests in several crops for spruce bud worm (Morris 1954); cabbage looper (Harcourt 1966; Shepard 1973); okra fruit borer (Rai et al.1982); brown plant hopper in rice (Shepard et al. 1986; Kumar 1996); lepidopteran caterpillars in fresh market collard (Smith and Shepard 2004).

In plans A and B sampling is continued until the infestation is either below the lower, or above the upper decision lines. Similarly, the cumulative infestation for different samples drawn can be compared numerically with the values presented in Table 1, before arriving at a decision to initiate area-wide management of *R. ferrugineus*. For example, in plan A and plan B if the cumulative number of infested palms in one hectare is zero out of 150 palms then area-wide management is not required, while on the other hand if the cumulative number of infested palms in the same area for plans A and B is 6 or 5, respectively then area-wide management is essential. Similarly, if the infestation level in plan A for one hectare is between zero and five palms, then additional sampling is required, while under plan B for the same area, if the infestations recorded are between zero and four, then no decision can be made by sampling one hectare and an additional 150 palms will have to be sampled. This type of sampling in sequence will continue until an accurate decision to either implement or not to implement area-wide management of *R. ferrugineus* can be made (Table 1 and Figure 1).

These sampling plans can also be used to assess the performance of *R. ferrugineus*-IPM programmes that are already in progress. Pheromone based *R. ferrugineus* -IPM programmes have been used to successfully manage the pest on coconut in Sri Lanka and India (Rajapakse et al.1998; Faleiro 2005). However, there is no information on the duration such programmes need to be pursued. Carrying on with *R. ferrugineus*-IPM in the field when not required would be unnecessarily expensive. If upon sampling a given operational area where *R. ferrugineus*-IPM is in progress and it is repeatedly ascertained that the cumulative infestation is below the lower limit (Table 1), then the ongoing *R. ferrugineus*-IPM programme could be either called off or scaled down, maintaining only monitoring/surveillance activities for that particular operational area and diverting valuable resources where the pest is more severe, where the cumulative number of infested palms is persistently above the upper limit (Table 1). If however, infestation levels for a given area are between the lower and upper limits, then the *R. ferrugineus*-IPM programme in progress will have to be continued. This is unlike decision making for initiating area-wide management of *R. ferrugineus*, when no decision can be made at infestation levels that are between the lower and upper limits of the sampling plans presented in Table 1. As mentioned previously, for implementing area-wide management of *R. ferrugineus* additional areas in units of 150 palms will have to be sampled at such intermediate infestation levels until a definite decision on initiating area-wide management can be made. Sequential sampling plans include the predetermined accuracy and action threshold levels (Onsager 1976) and hence assist in making a realistic assessment of on going IPM programmes. In coconut, the use of pheromone traps baited with food to mass trap the adult population in endemic plantations is a vital component of the IPM strategy for area-wide management of *R. ferrugineus* (Rajapakse *et al*. 1998; Faleiro 2005). A recent study involving area-wide management of *R. ferrugineus* in coconut plantations along the East coast of India has shown that mass trapping of the pest over a period of 18 months between January, 2005 to July, 2006 reduced infestation levels in two villages from 1.6 and 2.7 per cent to 0.1 and 0.5 per cent, respectively (Sujatha et al. 2006).

**Table 1.**
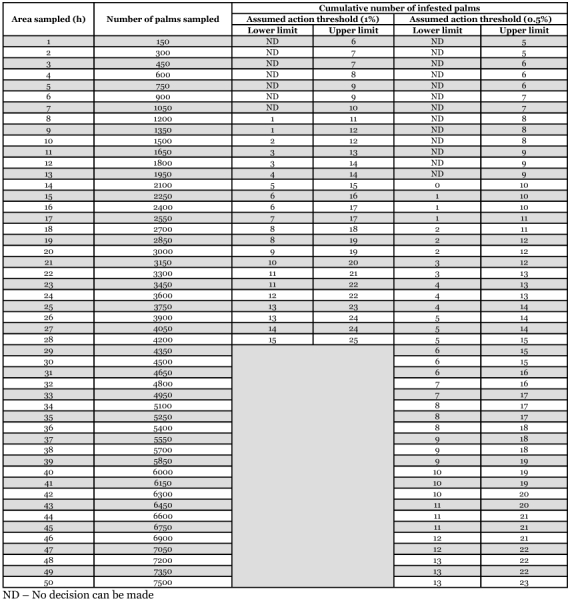
Sequential sampling table for initiating wide-area management of *R. ferrugineusin* coconut.

**Figure 2.**
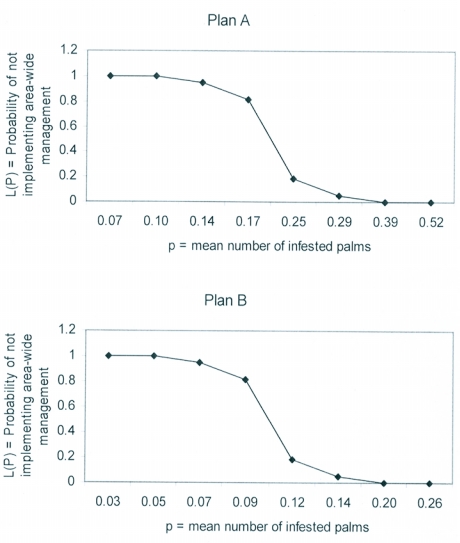
Operating characteristic (OC) curves for the sequential sampling plans of *Rhynchophorus ferrugineus* in coconut.

The OC curves for plans A and B of this study give the probability L (p) of accepting the hypothesis (i.e. not implementing area-wide management of *R. ferrugineus*) for a range of infestation means. As indicated in Figure 2 in both plans the probability of not accepting the hypothesis is high at low infestation means, with the reverse being true as infestation increases. This proves the accuracy of the plans developed. However, as shown by the simulation tools provided by Binns et al. (2000) at http://www.nysaes.cornell.edu/ent/faculty/nyrop/cpdm, a perfect OC curve would have a horizontal line at low infestation means (y=1) followed by a vertical drop at the threshold with a subsequent horizontal line to infinity at high infestation means (y=0).

**Figure 3.**
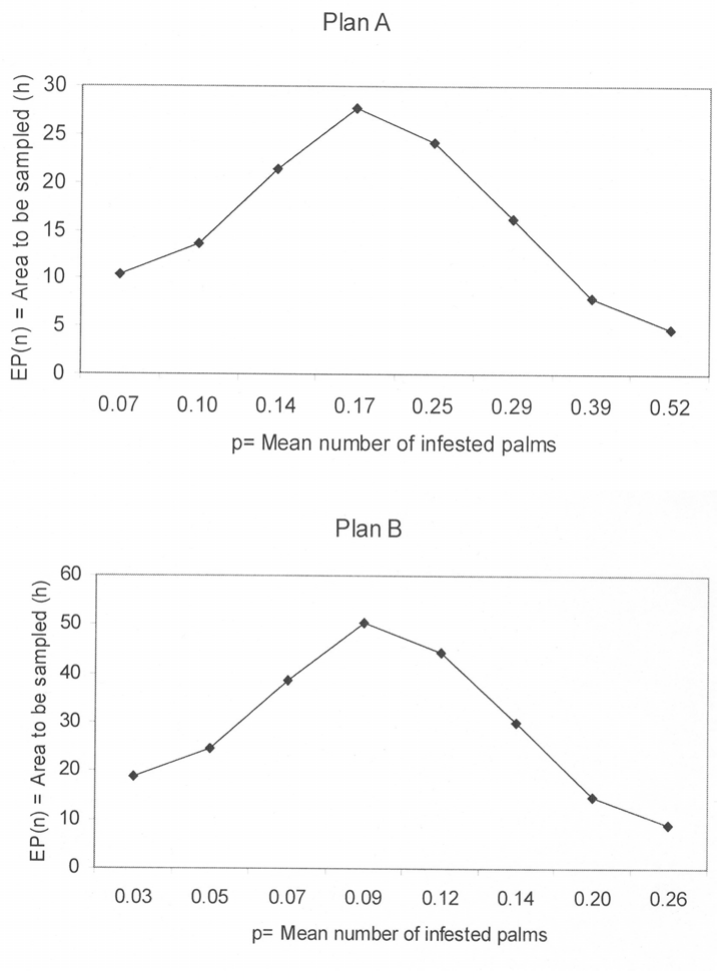
Average sample number (ASN) curves for the sequential sampling plans of *Rhynchophorus ferrugineus* in coconut.

Further, the ASN curves presented in Figure 3 reveal that at low and high infestation levels lower number of samples are required, while at medium infestation levels more samples are needed, which again shows the accuracy of the sampling plans devised. Similar OC and ASN curves have been developed to test the accuracy and visualize the performance of sequential sampling plans for several insect pests in the past (Morris 1954; Waters 1955; Harcourt 1966; Rai et al. 1982; Kumar 1996). The significance of OC and ASN curves in sequential sampling to evaluate the accuracy of classifying the infestation and number of sample required has been outlined by Nyrop and Binns (1991) and Binns and Nyrop (1992).

According to Ruesink and Kogan (1974) when sequential sampling plans are open-ended, sampling may continue indefinitely. This can happen at medium levels of infestation. To over come this problem of continuous open-ended sampling, the maximum number of samples computed through the ASN curves forms the basis to arrive at a cut-off point at which sampling should be stopped and a decision to implement area-wide management of *R. ferrugineus* should be taken. As per the ASN curves in this study we propose that sampling should be stopped at 28 hectare (4200 palms) for plan A, while for plan B the cut-off point recommended is 50 hectare (7500 palms).

## Conclusion

The sampling plans devised in this paper form the basis of rapidly and accurately classifying infestation levels due to *R. ferrugineus* in coconut and provide pest managers a valuable tool to confidently decide on initiating area-wide management of *R. ferrugineus* in coconut plantations of India, besides assisting assessment of the impact of on going management programmes thereby optimizing the use of resources available.

## Abbreviations

ASN -: average sample number
OC -: operating characteristic

## References

[bibr01] Abraham VA, Faleiro JR, Al-Shuaibi MA, Prem Kumar T (2000). A strategy to manage red palm weevil *Rhynchophorus ferrugineus* Oliv. on Date Palm *Phoenix dactylifera* L.- Its successful implementation in Al-Hassa, Kingdom of Saudi Arabia.. *Pestology*.

[bibr02] Abraham VA, Kurian C (1975). An integrated approach to the control of *Rhynchophorus ferrugineus* F. the red weevil of coconut palm. *Proceedings, 4th Session of the FAO Technical Working Party on Coconut Production, Protection and Processing* 14–25. September..

[bibr03] Abraham VA, Abdulla Koya KM, Kurian C (1989). Integrated management of red palm weevil (*Rhynchophorus ferrugineus* Oliv.) in coconut gardens.. *Journal of Plantation Crops*.

[bibr04] Binns MR, Nyrop JP (1992). Sampling insect populations for the purpose of IPM decision making.. *Annual Review of Entomology*.

[bibr05] Binns MR, Nyrop JP, Van der Werf W (2000). *Sampling and monitoring for crop protection decision making*..

[bibr06] Bliss CI, Owen ARG (1958). Negative binomial distribution with a common ‘K’.. *Biometrika*.

[bibr07] El-Ezaby F, Khalifa O, El-Assal A.Rahman, Al-Afifi MA, Al-Sharif Al-Badawy A (1998). Integrated Pest Management for the control of red palm weevil in the UAE Eastern region, Al-Ain.. Proceedings, First International conference on date palmsMarch, 1998269-281. Al-Ain, UAE.

[bibr08] Faleiro JR (2006a). Insight into the management of red palm weevil *Rhynchophorus ferrugineus* Olivier: Based on experiences on coconut in India and date palm in Saudi Arabia.. *Fundación Agroalimed*. Jornada Internacional sobre el Picudo Rojo de las Palmeras..

[bibr09] Faleiro JR (2006b). A review of the issues and management of red palm weevil *Rhynchophorus ferrugineus* (Coleoptera : Rhynchophoridae) in coconut and date palm during the last one hundred years.. *International Journal of Tropical Insect Science*.

[bibr10] Faleiro JR, Ashok Kumar J, Rangnekar PA (2002). Spatial distribution of red palm weevil *Rhynchophorus ferrugineus* Oliv. (Coleoptera : Cuculionidae) in coconut plantations.. *Crop Protection*.

[bibr11] Faleiro JR (2005). Pheromone technology for the management of red palm weevil *Rhynchophorus ferrugineus* (Olivier) (Coleoptera : Rhynchophoridae) -A key pest of coconut.. *Technical Bulletin No.4, ICAR Research Complex for Goa*..

[bibr12] Hallett RH, Gries G, Borden JH, Czyzewska E, Oehlschlager AC, Pierce HD, Angerilli NPD, Rauf A (1993). Aggregation pheromones of two Asian palm weevils, *Rhynchophorus ferrugineus* and *R. vulneratus*.. *Naturwissenschaften*.

[bibr13] Harcourt DG (1966). Sequential sampling plan for use in control of cabbage looper on cauliflower.. *Journal of Economic Entomology*.

[bibr14] Ives WGH, Warren GL (1965). Sequential sampling plans for white grubs.. *Canadian Entomologist*.

[bibr15] Kumar JA (1996). Sampling of Insect Populations-A Statistical Study. *Master of Science Thesis*..

[bibr16] Knipling EF (1992). Principles of insect parasitism analyzed from new perspectives: Practical implications for regulating insect populations by biological means. *Agriculture Handbook 693*..

[bibr17] Lindquist DA, Tan HK (1998). Pest management strategies: area-wide and conventional. *Area-wide control of fruit flies and other insect pests*.

[bibr18] Mathew MT (2004). Coconut industry in India: An overview.. *Indian Coconut Journal*.

[bibr19] Mumford JD, Tan HK (1998). Economics of area-wide pest control. *Area-wide control of fruit flies and other insect pests*.

[bibr20] Morris RF (1954). A sequential sampling technique for spruce bud worm egg surveys.. *Canadian Journal of Zoology*.

[bibr21] Nyrop JP, Binns MR, Pimentel D (1991). Quantitative methods for designing and analyzing sampling programme for use in pest management. CRC *handbook of pest management in agriculture*.

[bibr22] Nirula KK (1956). Investigation on the pests of coconut palm, Part-IV. *Rhynchophorus ferrugineus*.. *Indian Coconut Journal*.

[bibr23] Oakland GB (1950). An application of sequential analysis to white fish sampling.. *Biometrics*.

[bibr24] Onsager JA (1976). The rationale of sequential sampling with emphasis on its use in pest management.. *USDA Technical Bulletin No 1526*..

[bibr25] Rai S, Faleiro JR, Vasisht AK (1982). Sequential sampling plan for okra fruit bore, *Earias vittella* Fab.. *Annals of Agricultural Research*.

[bibr26] Rajapakse CNK, Gunawardena NE, Perera KFG (1998). Pheromone baited trap for the management of red palm weevil *Rhynchophorus ferrugineus* F. (Coleoptera: Curculionidae) population in coconut plantations.. *Cocos*.

[bibr27] Ruesink WG, Kogan M, Metcalf RL, Luckman WH (1974). The quantitative basis of pest management : Sampling and measuring. *Introduction to insect pest management*.

[bibr28] Shepard M (1973). A sequential sampling plan for treatment decision on the cabbage looper on cabbage.. *Environmental Entomology*.

[bibr29] Shepard M, Ferrer ER, Kenmore PE, Sumangil JP (1986). Sequential sampling: plant hoppers in rice.. *Crop protection*.

[bibr30] Smith JP, Shepard BM (2004). A binomial sequential sampling plan using a composite threshold for caterpillar management in fresh market collard.. *Journal of Agricultural and Urban Entomology*.

[bibr31] Southwood TRE, Henderson PA (2000). *Ecological methods* 3..

[bibr32] Suman CL, Wahi SD (1981). Sequential sampling plan for onion thrips, *Thrips tabaci* (L).. *Entomon*.

[bibr33] Sujatha A, Chalapathirao Rao NBV, Rao DVR (2006). Field evaluation of two pheromone lures against red weevil, (*Rhynchophorus ferrugineus* Oliv.) in coconut gardens in Andhra Pradesh.. *Journal of plantation crops*.

[bibr34] Soroker V, Blumberg D, Haberman A, Hamburger-Rishad M, Reneh S, Talebaev S, Anshelevich L, Harari AR (2005). Current status of red palm weevil infestation in date palm plantations in Israel.. *Phytoparasitica*.

[bibr35] Stern VM, Smith RF, Van Den Bosch R, Hagen KS (1959). The integrated control concept.. *Hilgardia*.

[bibr36] Thampan PK (1981). *Hand book of coconut palm*..

[bibr37] Vidhyasagar PSPV, Mohammed Hagi, Abozuhairah RA, Al-Mohanna OE, Al-Saihati AA (2000). Impact of mass pheromone trapping on red palm weevil adult population and infestation level in date palm gardens of Saudi Arabia.. *Planter*.

[bibr38] Waters WE (1955). Sequential sampling in forest insect surveys.. *Forest Science*.

[bibr39] Waters WE (1959). A quantitative measure of aggregation in insects.. *Journal of Economic Entomology*.

[bibr40] Wald A (1947). *Sequential analysis*..

[bibr41] Yu DJ, Xia LL, Fen WS, Xue WW (2005). Spatial pattern of *Celypha pseudolaxicola* larvae population within different parts of crown and its application.. *Acta Agriculturae Shanghai*.

[bibr42] Yu R, Leung P (2006). Optimal pest management : A reproductive pollutant perspective.. *International Journal of Pest Management*.

